# Supplementary material for: Modeling the Lassa fever outbreak synchronously occurring with cholera and COVID-19 outbreaks in Nigeria 2021: A threat to Global Health Security
Source: PLOS Glob Public Health. 2023 May 16;3(5):e0001814. doi: 10.1371/journal.pgph.0001814 (PMC10187896; doi:10.1371/journal.pgph.0001814)
Supplement: S1 Table — (DOCX) [file pgph.0001814.s001.docx]

Appendix 1: Nigeria CDC case definitions for Lassa fever, COVID-19, and cholera, 2021

**Source:** Nigeria CDC, Emergency Operations Centre, Cholera Technical Working Group

| **Disease** | **Case definitions** |
| --- | --- |
| Lassa fever | - **Suspected case:** Any individual presenting with one or more of the following: malaise, fever, headache, sore throat, cough, nausea, vomiting, diarrhea, myalgia, chest pain, hearing loss, and either (a) History of contact with excreta or urine of rodents (b) History of contact with a probable or confirmed Lassa fever case within a period of 21 days of onset of symptoms OR Any person with inexplicable bleeding/hemorrhagia. - **Confirmed case:** Any suspected case with laboratory confirmation (positive IgM antibody, PCR or virus isolation). - **Probable case:** Any suspected case (see definition above) who died or absconded without collection of specimens for laboratory testing. - **Contact:** Anyone who has been exposed to an infected person, or to an infected person’s secretions, excretions, or tissues within three weeks of last contact with a confirmed or probable case of Lassa fever. |
| COVID-19 | - **Suspected case:** Any person (including severely ill patients) presenting with fever, cough, or difficulty in breathing AND who within 14 days before the onset of illness, had any of the following exposures: (a) History of travel to and more than 24 hours transit through any high-risk country with widespread community transmission of SARS-CoV-2 OR (b) Close contact with a confirmed case of COVID-19 OR (c) Exposure to a healthcare facility where COVID-19 case(s) have been reported. - **Probable case:** Any suspect case (a) For whom testing for COVID-19 is Indeterminate test results OR (b) For whom testing was positive on a pan-coronavirus assay OR (c) Where samples were not collected before the demise of a suspect case. - **Confirmed case:** Any person with laboratory confirmation of SARS-CoV-2 infection with or without signs and symptoms. |
| Cholera | - **Suspected case:** Any patient aged ≥2 years with acute watery diarrhea and severe dehydration or dying from acute waterydiarrheaa with or without vomiting OR in areas where a Cholera outbreak is declared, any person presenting with or dying from acute watery diarrhea with or without vomiting. - **Confirmed case:** A suspected case in which *Vibrio cholerae* O1 or O1 39 has been isolated in the stool by culture. |
