# Supplementary material for: Modeling the Lassa fever outbreak synchronously occurring with cholera and COVID-19 outbreaks in Nigeria 2021: A threat to Global Health Security
Source: PLOS Glob Public Health. 2023 May 16;3(5):e0001814. doi: 10.1371/journal.pgph.0001814 (PMC10187896; doi:10.1371/journal.pgph.0001814)
Supplement: S1 Data — (DOCX) [file pgph.0001814.s002.docx]

**Appendix 2: Case numbers for Lassa fever, COVID-19, and Cholera for Nigeria 2021**

| **Week** | **Suspected Lassa fever cases** | **confirmed Lassa fever cases** | **probable Lassa fever cases** | **Lassa fever deaths** | **Number of states** | **Month** | **Cholera** | **COVID-19** |
| --- | --- | --- | --- | --- | --- | --- | --- | --- |
| 1 | 109 | 9 | 0 | 2 | 4 | 1 |  | 9940 |
| 2 | 117 | 14 | 0 | 2 | 3 | 1 |  | 10300 |
| 3 | 129 | 17 | 0 | 3 | 3 | 1 |  | 11179 |
| 4 | 153 | 14 | 0 | 2 | 6 | 1 |  | 9676 |
| 5 | 107 | 12 | 0 | 1 | 3 | 2 |  | 8506 |
| 6 | 91 | 17 | 0 | 6 | 3 | 2 |  | 6606 |
| 7 | 113 | 19 | 1 | 3 | 6 | 2 |  | 5720 |
| 8 | 140 | 34 | 0 | 6 | 10 | 2 |  | 3583 |
| 9 | 107 | 25 | 0 | 1 | 6 | 3 |  | 2878 |
| 10 | 145 | 30 | 0 | 6 | 7 | 3 |  | 2122 |
| 11 | 136 | 32 | 0 | 4 | 8 | 3 |  | 1080 |
| 12 | 84 | 7 | 0 | 3 | 2 | 3 | 1746 | 856 |
| 13 | 78 | 3 | 1 | 0 | 1 | 3 |  | 602 |
| 14 | 43 | 8 | 0 | 1 | 4 | 4 |  | 598 |
| 15 | 43 | 6 | 0 | 0 | 2 | 4 |  | 440 |
| 16 | 59 | 7 | 0 | 2 | 4 | 4 |  | 486 |
| 17 | 73 | 8 | 0 | 3 | 4 | 4 |  | 462 |
| 18 | 61 | 10 | 0 | 0 | 4 | 5 |  | 238 |
| 19 | 61 | 1 | 0 | 1 | 1 | 5 |  | 290 |
| 20 | 83 | 8 | 0 | 0 | 2 | 5 |  | 310 |
| 21 |  | 5 |  |  | 2 | 5 |  | 210 |
| 22 | 52 | 6 | 0 | 1 | 2 | 6 |  | 200 |
| 23 |  | 17 |  |  | 2 | 6 |  | 180 |
| 24 |  | 6 |  |  | 2 | 6 |  | 192 |
| 25 | 65 | 13 | 0 | 1 | 2 | 6 | 1725 | 209 |
| 26 | 67 | 3 | 0 | 0 | 2 | 7 | 1865 | 392 |
| 27 | 69 | 7 | 0 | 4 | 2 | 7 | 1885 | 693 |
| 28 | 70 | 5 | 0 | 0 | 2 | 7 |  | 966 |
| 29 | 66 | 4 | 0 | 1 | 3 | 7 | 650 | 1579 |
| 30 |  | 12 |  |  | 2 | 8 | 1162 | 2700 |
| 31 | 65 | 4 | 0 | 1 | 2 | 8 | 3781 | 3771 |
| 32 |  | 2 |  |  | 2 | 8 | 2984 | 4417 |
| 33 | 58 | 2 | 0 | 0 | 2 | 8 | 3098 | 4520 |
| 34 | 83 | 9 | 0 |  | 2 | 8 | 2127 | 4322 |
| 35 | 74 | 1 | 0 | 0 | 0 | 9 | 1677 | 4166 |
| 36 | 60 | 4 | 0 | 3 | 2 | 9 | 1182 | 3640 |
| 37 | 73 | 1 | 0 | 0 | 1 | 9 | 1825 | 2647 |
| 38 | 71 | 9 | 0 | 1 | 3 | 9 | 633 | 2658 |
| 39 | 51 | 3 | 0 | 0 | 2 | 10 | 1063 | 1682 |
| 40 | 63 | 4 | 0 | 1 | 1 | 10 | 535 | 1556 |
| 41 | 67 | 7 | 0 | 0 | 3 | 10 | 417 | 1604 |
| 42 | 81 | 8 | 0 | 0 | 4 | 10 | 254 | 1162 |
| 43 | 77 | 2 | 0 | 0 | 2 | 10 | 409 | 1501 |
| 44 | 72 | 7 | 0 | 0 | 3 | 11 | 392 | 752 |
| 45 | 113 | 8 | 0 | 0 | 4 | 11 | 148 | 434 |
| 46 | 108 | 9 | 0 | 1 | 3 | 11 | 440 | 442 |
| 47 | 91 | 3 | 1 | 0 | 3 | 11 | 217 | 530 |
| 48 | 84 | 4 | 0 | 0 | 4 | 12 | 201 | 503 |
| 49 | 123 | 10 | 1 | 3 | 3 | 12 | 227 | 2859 |
| 50 | 190 | 10 | 0 | 3 | 4 | 12 | 285 | 6406 |
| 51 | 193 | 29 | 0 | 3 | 4 | 12 | 280 | 13674 |
| 52 | 174 | 28 | 1 | 3 | 6 | 12 | 52 | 5889 |
