# Supplementary material for: Modeling the Lassa fever outbreak synchronously occurring with cholera and COVID-19 outbreaks in Nigeria 2021: A threat to Global Health Security
Source: PLOS Glob Public Health. 2023 May 16;3(5):e0001814. doi: 10.1371/journal.pgph.0001814 (PMC10187896; doi:10.1371/journal.pgph.0001814)
Supplement: Attachment — Submitted filename: PGPH-D-23-00263_Manuscript response letter (03.20).docx [file pgph.0001814.s003.docx]

Nancy Tahmo

College of Public Health

University of Nebraska Medical Center

68198-4365 Nebraska Medical Center

March 20, 2023

Dear Editor-in-Chief

Thank you for sharing the reviewer’s comments on the manuscript titled “Modeling the Lassa fever outbreak synchronously occurring with cholera and COVID-19 outbreaks in Nigeria 2021: a threat to Global Health Security” for consideration by PLOS Global Public Health. We have addressed the comments point-by-point, highlighted in the manuscript:

1. *Please, provide details on the disease surveillance tools employed to detect cases of the individual diseases with context about the reliability of case data.* - The case definitions have been mentioned on lines 89-91.
2. *Please, review your reference list to ensure that it is complete and correct. If you have cited papers that have been retracted, please include the rationale for doing so in the manuscript text, or remove these references and replace them with relevant current references. Any changes to the reference list should be mentioned in the rebuttal letter that accompanies your revised manuscript. If you need to cite a retracted article, indicate the article’s retracted status in the References list and also include a citation and full reference for the retraction notice.* – References have been reviewed.

We appreciate your expert input. Please, address all correspondence about this manuscript to [nancytahmo@gmail.com; nancy.tahmo@unmc.edu](mailto:nancytahmo@gmail.com;%20nancy.tahmo@unmc.edu).

Thank you for your consideration.

Sincerely,

Nancy Tahmo
